# Optimization of Nano-SiO_2_/Tea Polyphenol/Pullulan Edible Composite Films for Postharvest Preservation of Cherry Tomatoes

**DOI:** 10.3390/foods14193386

**Published:** 2025-09-30

**Authors:** Peng Huang, Jie Ding, Yu Han, Ling Gong, Fang Wu, Yaowen Liu, Pinyao Zhao, Zuying Yang, Lin Ye, Shanshan Zhou, Wen Qin

**Affiliations:** 1 Department of Quality Management & Inspection, Yibin University, 8 Jiusheng Road, Yibin 644000, China; 15908207395@163.com (Y.H.); glingdeemail@163.com (L.G.); zhaopy@yibinu.edu.cn (P.Z.); 2 College of Food Science, Sichuan Agricultural University, 46 Xinkang Road, Ya’an 625014, China; yaowenliu@sicau.edu.cn; 3 College of Food Science and Technology, Sichuan Tourism University, 459 Hongling Road, Chengdu 610100, China; dingjiedream@163.com; 4 Sichuan Higher Education Engineering Research Center for Agri-Food Standardization and Inspection, Yibin University, 8 Jiusheng Road, Yibin 644000, China; 18080988236@163.com (F.W.); y13440051310@sina.com (Z.Y.); yelinlyn1992@gmail.com (L.Y.); dearzhoushanshan@163.com (S.Z.)

**Keywords:** pullulan-based coating, amorphous nano-silica, Box–Behnken design, ternary composite, food safety, postharvest quality, cherry tomato

## Abstract

Edible composite coatings represent an alternative approach to reducing postharvest losses and extending the shelf life of perishable fruits. This study developed a nano-biopolymer coating by integrating pullulan (PUL), nano-silica (Nano-SiO_2_), and tea polyphenols (TP) to retard deterioration in cherry tomatoes (*Solanum lycopersicum* var. *cerasiforme*). Optimized through response surface methodology (0.06% Nano-SiO_2_, 0.1% TP, 1.8% PUL, 0.77% glycerol), the resulting Nano-SiO_2_/PUL/TP composite film showed improved barrier properties (water vapor permeability, WVP: 0.2063 g·mm·m^−2^·h^−1^·kPa^−1^) and increased mechanical strength (tensile strength, TS: 2.62 MPa; elongation at break, EB: 67.67%), which may be attributed to a homogeneous microstructure stabilized via intermolecular hydrogen bonding. The composite coating exhibited significant (*p* < 0.05) antioxidant activity (59.04% DPPH·scavenging) compared to the PUL film (1.17%) and showed efficacy against S. aureus. When applied to cherry tomatoes stored at 4 °C for 15 days, the coating contributed to improved postharvest quality by reducing weight loss (−27.6%) and decay incidence (−32.3%), delaying firmness loss (2.40 vs. 0.54 N in uncoated group, CK), suppressing respiration rate (−38.8%), and enhancing the retention of total acidity (+9.7%), vitamin C (+49.6%), and total soluble solids (+48.6%) compared to the CK (*p* < 0.05). Principal component analysis supported sensory evaluation results, indicating the coating helped maintain sensory quality (scores > 6.0) and commercial value while extending shelf life from 9 to 15 days. These results suggest that the Nano-SiO_2_/TP/PUL composite coating may serve as a preservative for extending the shelf-life of cherry tomatoes by effectively reducing decay and mitigating quality degradation.

## 1. Introduction

Maintaining the postharvest quality of fresh produce is essential for supporting global supply chains and promoting sustainable economic growth [1,2,3]. Cherry tomatoes (*Solanum lycopersicum* var. *cerasiforme*), recognized by the FAO as a priority crop due to their high nutritional value—including β-carotene, vitamins, and minerals—and bioactive properties such as antioxidant and anticancer activities, face significant postharvest challenges [4,5]. Their inherently high respiration rate, combined with high water content (>90%), accelerates physiological deterioration, resulting in severe moisture loss (exceeding 30%) and widespread decay during storage and transport [6,7,8]. While conventional approaches such as chemical treatments and refrigeration offer partial mitigation, increasing sustainability concerns have shifted research focus toward eco-friendly, biopolymer-based edible coatings [9,10]. This reorientation highlights high-performance eco-composite films as a continually relevant research frontier, essential not only for significantly extending shelf life but also for supporting fundamental circular economy principles [2,11,12].

Edible films represent a sustainable preservation strategy that utilizes natural, biodegradable, and food-safe materials to extend shelf life [13,14]. When used within approved limits, many biopolymers are widely recognized as GRAS (Generally Recognized As Safe) by the U.S. FDA for specific food applications [15]. These materials form semi-permeable barriers through immersion, spraying, or brushing [15,16,17], allowing precise control over gas exchange and water vapor transmission, which effectively inhibits respiratory metabolism while helping maintain product quality [18]. Among polysaccharide matrices, pullulan [19], chitosan [20], pectin [9], and alginate [21] are particularly notable due to their biocompatibility, cost-effectiveness, and commercial viability.

Pullulan (PUL), an extracellular polysaccharide produced by *Aureobasidium* pullulans via starch/sugar fermentation, is used as a low-calorie food additive, adhesive, thickener, and microencapsulant. Its exceptional film-forming capacity, characterized by non-toxicity, odorlessness, biocompatibility, flexibility, and low oxygen permeability, have accelerated packaging applications [19,22]. However, pure PUL coatings exhibit inherent limitations, including inadequate mechanical strength, high hydrophilicity, substantial production costs, and limited product protection [23,24]. Consequently, research prioritizes functional additive integration to augment performance, establishing PUL matrices as bioactive carriers for antimicrobial, antioxidant, and anti-browning agents [25,26]. Zhang et al. [27] reported that ZnO nanoparticles within polyvinyl alcohol/pullulan/ZnO nanoparticles (PVA/PUL/ZnO-Nps) composites significantly reduced light transmittance while enhancing water contact angle, moisture barrier, mechanical strength, and antimicrobial activity, thereby extending kiwifruit shelf-life. Similarly, PUL films incorporating curcumin and orange essential oil nanoemulsions exhibited optimal mechanical properties and minimal water vapor permeability, prolonging oil retention, reducing strawberry weight loss, and decreasing spoilage incidence by 44% [28].

Tea polyphenols (TP), naturally occurring polyphenols abundant in tea leaves, possess phenolic hydroxyl groups acting as natural crosslinkers that reinforce composite film integrity [29,30]. Li et al. [31] demonstrated that TP-loaded corn zein/apple pectin films possess excellent morphology, water resistance, antibacterial/antioxidant capacity, and preservation efficacy for fresh walnuts. Similarly, Benlloch-Tinoco et al. [21] reported enhanced physical, mechanical, and barrier properties in alginate films containing green tea polyphenols. Nano-silicon dioxide (Nano-SiO_2_), an amorphous nanomaterial with a high surface area-to-volume ratio, is widely employed in the food industry owing to its low toxicity, biocompatibility, optical transparency, and chemical stability [32]. SiO_2_ is commonly used as a food additive and is approved as E551 in the European Union for its anti-caking and flow-enhancing properties. Although E551 includes synthetic amorphous silica (SAS), the European Food Safety Authority (EFSA) notes that nanoforms require specific safety assessments [33]. The U.S. FDA also classifies silica as GRAS but acknowledges that properties such as particle size may affect safety evaluations. In China, the GB 2760 standard permits the use of SiO_2_ as an anti-caking agent without explicitly distinguishing nanoforms, although ongoing evaluations are addressing nanospecific considerations [34]. As a sophisticated nanofiller, SiO_2_ significantly enhances the physicochemical properties of edible films [35,36]. Zhu et al. [32] showed that Si nanoparticle-reinforced polysaccharide films mitigated moisture loss in mushrooms while boosting retention of phenolics and vitamin C. Likewise, Zhang et al. [34] demonstrated that Nano-SiO_2_ promotes controlled release of bioactives in degradable packaging, thereby enhancing antioxidant and antimicrobial functionality.

Despite these advancements, a significant research gap remains in the systematic, RSM-guided co-optimization of amorphous nano-silica and tea polyphenol incorporation within a pullulan matrix, as well as in the subsequent validation of its efficacy on cherry tomatoes. To address this, the present study utilizes PUL as the matrix, optimizing the Nano-SiO_2_/TP/PUL formulation via response surface methodology, characterizing the microstructure using SEM and FTIR, and evaluating key physico-mechanical properties (including light transmittance, water vapor permeability, tensile strength, and elongation at break). Functional properties, such as antioxidant and antibacterial activities, are also systematically evaluated. Furthermore, the composite coating was applied to cherry tomatoes to assess its effects on fruit quality parameters during storage. Preservation outcomes were comprehensively evaluated using principal component analysis, establishing a theoretical foundation for the application of Nano-SiO_2_/TP/PUL edible films in extending fruit shelf life.

## 2. Materials and Methods

### 2.1. Materials and Reagents

Food-grade PUL and TP were obtained from Jiahe Xuri Food Industry Co., Ltd. (Suzhou, China). Nano-SiO_2_ with an average particle size of 30 nm was procured from Guangzhou Kangda Biotechnology Co., Ltd. (Guangzhou, China) in the form of a white fluffy powder. In October 2024, commercially mature cherry tomatoes (*Solanum lycopersicum* var. *cerasiforme* cv. ‘Qianxi’) were harvested from farms in Yibin, Sichuan Province, China. To ensure homogeneity, fruits with uniform color and size, and free from physical defects or pest infestation, were selected. All chemical reagents used were of analytical grade and purchased from Shanghai Aladdin Biochemical Technology Co., Ltd. (Shanghai, China)

### 2.2. Composite Film Preparation and Optimization

#### 2.2.1. Preparation of Nano-SiO_2_/TP/PUL Composite Film

The composite film was prepared following the method described by Zhang et al. [23] with slight modifications. Briefly, distilled water (100 mL) was preheated to 60 °C. Subsequently, PUL (2.0 g), TP (0.1 g), Nano-SiO_2_ (0.08 g, 30 nm), and glycerol (0.8 g) were added sequentially to the preheated aqueous phase under constant stirring at 1200 rpm for 30 min using a mechanical stirrer (LCA-MSC-2HD, Li Chen Technology Co., Ltd., Shanghai, China). The mixture was then subjected to ultrasonic treatment (25 kHz, 30 min) to remove bubbles (LC-BUC-32, Li Chen Technology Co., Ltd., China). After cooling to room temperature, the solution was brought to a final volume of 20 mL. A measured volume (20 mL) of the solution was poured onto preheated Petri dishes and dried in an electric thermostatic oven (GZX-9023MBE, Boxun Technology Co., Ltd., Shanghai, China) at 60 °C for 5 h. The resulting dried films were conditioned at room temperature, carefully peeled off, and stored in sealed polyethylene bags before further analysis [35].

#### 2.2.2. Single-Factor Experimental Design

The optimal concentrations of TP, Nano-SiO_2_, PUL, and glycerol were determined using a single-factor experimental design. The base formulation of the film-forming solution was established as PUL 2.0 g/100 mL, TP 0.1 g/100 mL, Nano-SiO_2_ 0.08 g/100 mL, and glycerol 0.8 g/100 mL. This baseline was selected based on preliminary experiments as the central point that produced a handleable film with balanced properties. Tensile strength (TS), elongation at break (EB), and water vapor permeability (WVP) were selected as indicators to evaluate the overall performance through membership degree analysis [37]. The influence of different concentrations of each component—TP (0.0, 0.05, 0.10, 0.15, and 0.20 g/100 mL), Nano-SiO_2_ (0.02, 0.05, 0.06, 0.08, and 0.10 g/100 mL), PUL (1.5, 1.75, 2.0, 2.25, and 2.5 g/100 mL), and glycerol (0.4, 0.6, 0.8, 1.0, and 1.2 g/100 mL)—on the properties of the composite films was investigated. All composite films were prepared following the procedure outlined in Section 2.2.1.

#### 2.2.3. Response Surface Experimental Design

Based on the results of the single-factor tests and comprehensive performance evaluation, a Box–Behnken design (BBD) was employed to construct a three-factor, three-level response surface methodology (RSM) model using Design-Expert 13 software (Stat-Ease, Inc., Minneapolis, MN, USA). With the objective of comprehensively optimizing the composite film properties, the concentrations of Nano-SiO_2_, PUL, and glycerol were chosen as independent variables, while the comprehensive performance score—derived from tensile strength, elongation at break, and water vapor permeability—was used as the response variable [23,38]. The factors and levels used in the response surface design are presented in Table 2.

### 2.3. Mechanical Properties of Edible Composite Films

Film specimens (40 × 10 mm) were secured between tensile grips of a texture analyzer (TA-XTC-18, Baosheng Co., Shanghai, China). Tensile tests were conducted with an initial gauge length of 30 mm and a crosshead speed of 1 mm/s [27]. The tensile strength (TS, MPa) and elongation at break (EB, %) were calculated using the following equations:

(1) $$TS=\frac{F}{W\times x}$$

where *F* is the tensile force (N), *W* is the width of the film sample (mm), and *x* is the thickness of the membrane sample (mm).

(2) $$EB(\%)=\frac{L}{L_{0}}\times 100\%$$

*L* is the elongation (mm) of the diaphragm when it breaks; *L*_0_ is the initial length (mm) of the diaphragm.

### 2.4. Physical Properties of Edible Composite Films

#### 2.4.1. The Light Transmittance of Edible Composite Films

A rectangular specimen (10 × 30 mm) was cut from the composite film and affixed to the inner surface of a glass cuvette in perpendicular orientation to the light path. Light transmittance (LT) at 600 nm [39] was measured using a UV-Vis spectrophotometer (T6, Puxi Co., Beijing, China), with an empty cuvette used as a blank control.

#### 2.4.2. Water Vapor Permeability of Edible Composite Films

Water vapor permeability (WVP) was determined at 25 °C by sealing an intact film specimen over a triangular flask containing 15 mL of distilled water [40]. The assembly was hermetically sealed, allowed to equilibrate for 1 h, and the initial mass was recorded. The mass was then measured every 3 h over 9 h in a desiccator containing silica gel. The WVP (g·mm·m^−2^·h^−1^·kPa^−1^) was calculated using the following formula:

(3) $$WVP=\frac{\Delta m\times x}{\Delta t\times A\times \Delta P}$$

where Δ*m* = Mass change in the film (g), *x* = film thickness (mm), *A* = effective permeation area (m^2^), Δ*t* = time interval (h), and Δ*P* = water vapor partial pressure differential across the film at 25 °C (kPa).

### 2.5. Functional Properties of Composite Films

#### 2.5.1. Determination of Antioxidant Properties

A 12.50 mg sample of the film was dissolved in 10 mL of deionized water at 90 °C. After being cooled to room temperature, the solution was diluted to a final volume of 25 mL in a volumetric flask and equilibrated at room temperature for 30 min. A 2.5 mL aliquot of the supernatant was mixed with 2.5 mL of a 0.2 mmol/L ethanolic DPPH solution, which was prepared by dissolving 4.0 mg of DPPH in 50 mL of anhydrous ethanol. The mixture was incubated in the dark for 1 h. The absorbance was measured at 517 nm using a UV-Vis spectrophotometer, with anhydrous ethanol used as the blank [41]. The radical scavenging activity (RSA, %) was calculated using the following equation:

(4) $$P=\left(1-\frac{X_{1}-X_{2}}{X_{0}}\right)\times 100\%$$

where *P* = DPPH·scavenging activity (%), *X*_1_ = absorbance of supernatant–DPPH mixture, *X*_2_ = absorbance of supernatant–ethanol mixture (optional background correction), and *X*_0_ = absorbance of DPPH solution mixed with solvent (distilled water).

#### 2.5.2. Determination of the Antibacterial Property

The antibacterial activity of the composite film solution was evaluated against *Staphylococcus aureus* following the method described by Zhang et al. [23] with minor modifications. A 200 μL aliquot of the bacterial suspension, adjusted to approximately 10^8^ CFU/mL in saline, was evenly coated onto nutrient agar plates. Then, 150 μL of the Nano-SiO_2_/TP/PUL composite film solution (test sample) and the single-component PUL film solution (control sample) were added to separate sterile Oxford cups placed on the inoculated agar. The plates were incubated at 37 °C for 24 h, and the diameter of the inhibition zone was measured.

### 2.6. Structural Characterization of Films

#### 2.6.1. Scanning Electron Microscopy

The surface morphology of the composite films was examined using scanning electron microscopy (SEM). Samples were cut into 5 × 5 mm squares, mounted on aluminium stubs with conductive double-sided carbon tape, and sputter-coated with a 10 nm gold layer. Imaging was carried out at an accelerating voltage of 15 kV with a secondary electron detector. Representative images were acquired at 1500× magnification [1]. Scale bars were calibrated for dimensional accuracy in all micrographs.

#### 2.6.2. ATR-FTIR Spectra

Dried composite films were cryogenically ground into fine powder (<2 μm particle size) and homogenized with spectroscopic-grade potassium bromide (KBr) at a 1:100 (*w*/*w*) ratio. The mixture was thoroughly triturated in an agate mortar for 5 min and then compressed into 13 mm pellets under an 8-ton vacuum for 3 min. FTIR spectra were recorded using a spectrometer (Nicolet 5700, Varian, Santa Clara, CA, USA) continuously purged with dry nitrogen gas, acquiring 32 co-added scans over the range of 4000–400 cm^−1^ at a resolution of 4 cm^−1^ [42]. All spectra were background-corrected using pure KBr pellets.

### 2.7. Evaluation of the K Value for the Composite Films

The comprehensive performance of the Nano-SiO_2_/TP/PUL edible composite films was evaluated via fuzzy comprehensive assessment based on key physical properties [37]. The experimental data underwent fuzzy transformation, and the film performance scores were calculated according to the following functions:

For benefit-type indicators (TS, EB):

(5) $$M=\frac{X_{i}-X_{\min}}{X_{\max}-X_{\min}}$$

For negative indicators (WVP):

(6) $$M=1-\frac{X_{i}-X_{\min}}{X_{\max}-X_{\min}}$$

where *M* = film’s degree of sample, *Xi* = measured value of sample, *X_max_* = maximum value in dataset, and *X_min_* = minimum value in dataset.

Given the focus on mechanical and barrier properties, a weight vector Y = (0.3, 0.3, 0.4) was assigned to reflect the relative contribution of each criterion to comprehensive performance: membership grade *M*_1_ (TS), *M*_2_ (EB), and *M*_3_ (WVP). The composite performance score *K* was computed as follows:

(7) $$K=Y\cdot M^{T}=(0.3,\;0.3,\;0.3)\cdot {\left(M_{1},\;M_{2},\;M_{3}\right)}^{T}$$

### 2.8. Coating Application for the Storage of Cherry Tomatoes

#### 2.8.1. Coating Treatment for Cherry Tomato

Cherry tomatoes with uniform color and similar sizes, devoid of mechanical damage or pest infestation, were selected and randomly divided into five groups (*n* = 60 per group). Samples from the experimental groups (Table A1) were dipped in the coating solution for 15 min, air-dried at room temperature (25 ± 1 °C; 70 ± 2% RH), and stored at 4 ± 1 °C for 15 days. Quality parameters were evaluated at 3-day intervals. The control (CK) group was treated with distilled water.

#### 2.8.2. Determination of Weight Loss

For each treatment group, 10 cherry tomatoes were selected and weighed at storage initiation using an analytical balance (±0.01 g readability). Percentage weight loss was calculated using Equation (8):

(8) $$\mathrm{WLR}\;(\%)\;=\;\frac{M_{0}-M_{t}}{M_{0}}\;\times 100$$

where *M*_0_ represents initial mass (g) and *M_t_* = mass at day *t*.

#### 2.8.3. Determination of Decay Rate

The decay assessment protocol was adapted from the method described by Ali et al. [19] with modifications. At each sampling point, 10 fruits per group were used as replicates for evaluation. Fruits were visually inspected, and those showing visible damage, such as browning, rotting, or mold covering more than 5% of the surface area, were classified as decayed. The decay rate (%) was calculated as follows:

(9) $$\mathrm{DR}\;(\%)\;=\;\frac{D_{f}-D_{t}}{D_{f}}\;\times 100$$

where *D_t_* is the number of decayed cherry tomato fruits and *D_f_* is the total samples per group.

#### 2.8.4. Determination of Firmness

Fruit hardness was measured using a digital texture analyzer (GY-5A, Ai Pu, Weifang, China). Five fruits per group were randomly selected, and three measurements were taken along the equatorial region of each fruit [38]. The mean value per group was expressed as the representative firmness value (N, mean ± standard deviation).

#### 2.8.5. Determination of Total Soluble Content

The total soluble solids (TSS) content of cherry tomatoes was determined using a digital refractometer (Model AK002B, Aipu, Weifang, China; accuracy: ±0.1% Brix). For each treatment, three fruits were randomly selected and homogenized without filtration. The homogenate was centrifuged (8000× *g*, 10 min, 4 °C), and the resulting supernatant was immediately assayed in triplicate (*n* = 3) at 20 °C using a calibrated refractometer.

#### 2.8.6. Determination of Titratable Acid

Titratable acidity (TA) was determined according to the NaOH titration method described by Tsague Donjio et al. [38]. A 0.05 mol/L NaOH solution was standardized using potassium hydrogen phthalate (KHP, ≥99.95%). A quantity of 5.00 ± 0.01 g of cherry tomato pulp homogenate was placed in a 50 mL volumetric flask, diluted to the mark with deionized water, and shaken mechanically at 500 rpm for 10 min. After equilibrating for 30 min at 25 °C, the suspension was filtered under vacuum through Whatman No. 1 filter paper. A 20.0 mL portion of the filtrate was titrated with the standardized NaOH solution, using phenolphthalein indicator (1% *w*/*v* in ethanol), to a stable, faint pink endpoint that persisted for at least 30 s at 25 °C, with the endpoint verified using a pH meter. TA was calculated as follows:

(10) $$\mathrm{TA}\;(\%)\;=\;\frac{{c\;\times \;V}_{s}\;\times \;V_{t}\;\times f}{V_{S}\;\times \;m}\times 100$$

where *V_t_* = total extract volume (50 mL), *V_S_* aliquot volume (20 mL). *c* = NaOH concentration (mol/L), *V_t_* = titrant volume (mL), *m* = sample mass (g), and 0.067 = citric acid equivalent (g/mmol).

#### 2.8.7. Determination of VC

Vitamin C (VC) content was determined by 2,6-dichlorophenol indophenol (DCPIP) titration according to AOAC Official Method 967.21 [43], with minor modifications. Briefly, filtered juice (10 mL) obtained from cherry tomatoes was homogenized with an equal volume of 0.4% (*w*/*v*) oxalic acid solution. The mixture was titrated with a standardized DCPIP solution until a persistent light pink endpoint (lasting ≥ 15 s) was reached. The ascorbic acid concentration was calculated using the following formula:

(11) $$VC\;\left(mg/100\;g\right)=\frac{(V-V_{0})\times T\times A}{m}\times 100$$

where *V* = DCPIP volume consumed by sample (mL), *V*_0_ = DCPIP volume consumed by blank (mL), *T* = DCPIP titration factor (mg ascorbic acid/mL), *A* = dilution factor, and *m* = sample mass (g).

#### 2.8.8. Determination of Respiration Rate

Respiration rate (RR) was determined using the closed-chamber alkali absorption method [8,16]. For each treatment, twenty cherry tomatoes (total mass recorded to ±0.01 g using an analytical balance) were equilibrated at 25 ± 1 °C for 30 min. The samples were hermetically sealed in 5 L desiccators, each containing 10.0 mL of 0.4 mol·L^−1^ NaOH, placed in a center well. After incubating at 25 °C for 60 min, the alkali solution was quantitatively transferred and titrated with standardized 0.2 mol·L^−1^ oxalic acid, using phenolphthalein as an indicator (endpoint: persistent disappearance of pink color for ≥30 s). Blank titrations were performed using identical chambers without biological material. RR, expressed as mg CO_2_·kg^−1^·h^−1^, was calculated as follows:

(12) $$RR(mg/(Kg*h))=\frac{(v_{1}-v_{2})\times c\times 22}{m\times t}$$

where *V*_1_ = oxalic acid volume for blank (mL), *V*_2_ = oxalic acid volume for sample (mL), *c* = oxalic acid concentration (mol/L), *m* = sample mass (kg), *t* = incubation time (h), and 22 = CO_2_ molar mass/2.

#### 2.8.9. Sensory Evaluation

Sensory evaluation of coated cherry tomatoes was conducted by a ten-member trained panel (five males, five females) following established methodology [44,45]. Panelists assessed appearance, odor, and texture using a 10-point descriptive scale. Detailed scoring criteria are provided in Table A2.

### 2.9. Statistical Analysis

All data were collected from three biologically independent replicates and are presented as mean ± standard deviation. Experimental design and response surface optimization were carried out using Design-Expert^®^ software (Version 13.0, Stat-Ease Inc., Minneapolis, MN, USA). Data visualization and principal component analysis (PCA) were conducted using OriginPro^®^ (Version 2025, OriginLab Corporation, Northampton, MA, USA). Statistical significance (α = 0.05) was determined by one-way analysis of variance (ANOVA) followed by Tukey’s post hoc test in IBM SPSS Statistics (Version 26.0, IBM Corp., Armonk, NY, USA).

## 3. Results and Discussion

### 3.1. Single-Factor Test Results

#### 3.1.1. Influence of Nano-SiO_2_ for Composite Film

The incorporation of Nano-SiO_2_ (0.02–0.10% *w*/*v*) significantly enhanced the properties of the TP/PUL composite films (*p* < 0.05) by promoting uniform dispersion and forming an interconnected spatial network [42]. Optical analysis (Table 1) revealed a significant decrease in light transmittance with increasing Nano-SiO_2_ concentration, likely due to enhanced light scattering by the nanoparticles. Water vapor permeability (WVP) showed a strong negative correlation with Nano-SiO_2_ content (*p* < 0.05), suggesting that interfacial interactions, such as potential hydrogen bonding, between nanoparticles and matrix components may promote structural densification. This result is consistent with the findings reported by Chen et al., [46]. Mechanically, tensile strength (TS) peaked at 3.03 MPa at 0.06% *w*/*v* before declining, which may be attributed to nanoparticle aggregation, while elongation at break (EB) increased steadily to 44.67%. Higher Nano-SiO_2_ content (>0.06% *w*/*v*) may induce local stress concentrations that disrupt polymer bonds, leading to reduced tensile strength [34,47,48]. Fuzzy comprehensive evaluation confirmed optimal performance at 0.06% *w*/*v* (K = 0.64), establishing this concentration as the central parameter for response surface optimization to balance functionality with structural integrity across optical, barrier, and mechanical properties.

#### 3.1.2. Influence of PUL for Composite Film

PUL concentration significantly affected the properties of the composite films (Table 1). Light transmittance (LT) exhibited a positive correlation with PUL content (*p* < 0.05), which aligns with previous reports [27]. WVP reached a minimum value (0.17 g·mm·m^−2^·h^−1^·kPa^−1^) at 1.75% *w*/*v*, likely due to improved intermolecular compatibility and structural densification, effectively inhibiting water diffusion. Beyond this concentration, increased hydrophilicity mediated by hydroxyl groups resulted in higher WVP, owing to reduced molecular packing density. Lower PUL concentrations maximized elongation at break (EB) but compromised tensile strength (TS), whereas higher concentrations (2.0–2.5% *w*/*v*) enhanced film rigidity at the expense of flexibility—a trend consistent with composite studies reported by Wani et al. [22]. Fuzzy comprehensive evaluation identified optimal overall performance at 1.75% *w*/*v* (K = 0.72), establishing this level as the central parameter for response surface optimization.

#### 3.1.3. Influence of TP for Composite Film

Tea polyphenol (TP) concentration significantly influenced the properties of the composite films (Table 1). Light transmittance remained stable (86.51–90.15%), while WVP exhibited a U-shaped trend, reaching a minimum value (0.18 g·mm·m^−2^·h^−1^·kPa^−1^) at 0.15% *w*/*v*, likely due to reduced molecular gaps. Mechanical properties were optimized at 0.1% *w*/*v*, with tensile strength (TS) reaching 2.75 MPa (a 56.3% increase) and elongation at break (EB) reaching 50.33% (a 125.5% increase) compared to the control. At concentrations below 0.15% *w*/*v*, enhanced intermolecular interactions promoted structural densification; however, higher concentrations led to particle aggregation and surface cracking, which compromised both barrier and mechanical performance [49,50]. Fuzzy comprehensive evaluation confirmed optimal functionality at 0.1% *w*/*v* (K = 0.63), consistent with previously reported polyphenol–polymer interaction mechanisms [21].

#### 3.1.4. Influence of Glycerol for Composite Film

Glycerol concentration (0.4–1.2% *w*/*v*) significantly affected the properties of the composite films (*p* < 0.05, Table 1). Beyond 1.2% *w*/*v*, the films exhibited adhesion and peeling defects. Optimal performance was achieved at 0.8% *w*/*v*, resulting in maximum light transmittance (92.10%, 38.5% higher than the control) and minimum water vapor permeability (0.25 g·mm·m^−2^·h^−1^·kPa^−1^, 28.6% reduction). The hydrophilicity and low molecular weight of glycerol likely facilitated its penetration into the polymer matrix, while hydrogen bonding with polysaccharide chains reduced intermolecular forces, thereby increasing WVP [51,52]. Mechanically, glycerol disrupted the polymer network through chain insertion, decreasing tensile strength by 18.2% but increasing elongation at break by 124% compared to the control—consistent with typical plasticizer effects in polysaccharide-based films [39,53]. Fuzzy comprehensive evaluation confirmed peak overall performance at 0.8% *w*/*v* (K = 0.64), establishing this concentration as the central point for response surface optimization.

### 3.2. Optimization by Response Surface Methodology

#### 3.2.1. Establishment of the Regression Model

Preliminary single-factor experiments were conducted to evaluate four components. These experiments revealed that although tea polyphenol (TP) exhibited a measurable effect, its influence on critical response parameters was less pronounced than that of the other factors. This was quantified by the comprehensive performance score (K-value). Therefore, the TP concentration was fixed at 0.1% for the response surface methodology (RSM) study. This allowed the optimization to focus on the interactions among Nano-SiO_2_ (A), pullulan (PUL, B), and glycerol (C). This approach streamlined the experimental design. A Box–Behnken design (Design-Expert 13.0) was employed to optimize these three key parameters governing composite film performance. The experimental design and corresponding responses are summarized in Table 2. Regression analysis yielded a second-order polynomial model describing the relationship between the K-value and the component concentrations:

*K* = 0.7632 + 0.0237*A* + 0.0830*B* − 0.0669*C* − 0.0298*AB* + 0.0637*AC* + 0.0876*BC* − 0.1259*A*^2^ − 0.123*B*^2^ − 0.1528*C*^2^.

Response surface methodology established a statistically significant quadratic model (*p* < 0.01, Table 3) with excellent predictive accuracy, as indicated by an R^2^ value of 0.9897 and an adjusted R^2^ of 0.9764. The model’s adequacy was further confirmed by a non-significant lack-of-fit test (*p* > 0.05) and residual analysis, effectively mitigating concerns regarding overfitting. A strong correlation between predicted and actual values was observed (>97.6% agreement), and the insignificant lack-of-fit (*p* > 0.05) further validated model reliability [51]. Analysis of variance (ANOVA) revealed highly significant effects (*p* < 0.01) on the comprehensive performance index (K-value) from the linear terms of pullulan (B) and glycerol (C), quadratic terms (A^2^, B^2^, C^2^), and the BC interaction term. Significant effects (*p* < 0.05) were also observed for the linear term of Nano-SiO_2_ (A) and the AC interaction. Factor hierarchy analysis (B > C > A), based on F-values and regression coefficients, identified pullulan concentration as the dominant factor affecting film performance, with glycerol exerting a moderate influence and Nano-SiO_2_ showing minimal impact [52,54]. The validated model provides an effective tool for optimizing the preparation of Nano-SiO_2_/TP/pullulan composite films.

**Figure 1:**
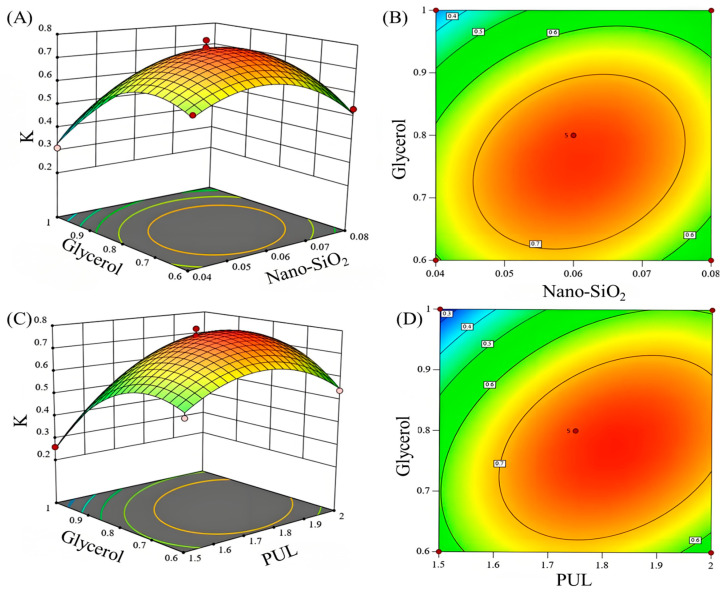
Analysis of the interaction effects on the performance of composite films through 3D graphs and contour plots. Note: (**A**,**B**) represent the interaction effect of the addition amounts of Nano-SiO_2_ and glycerol. (**C**,**D**) represent the interaction effect of the addition amounts of glycerol and PUL. *K* value represent comprehensive evaluation.

The significance of factor interactions on the response values was determined by analyzing the curvature of the response surfaces, which manifested as follows: steep gradients accompanied by tight elliptical contours indicated strong interactive effects, while gentle slopes with diffuse circular contours reflected weaker influences. As shown in Figure 1, pronounced curvature and elliptical contour distributions were observed for both the Nano-SiO_2_/glycerol (A–C) and PUL/glycerol (B–C) interactions, indicating that these factor interactions significantly affected the comprehensive performance score (K) of the composite films, thereby validating the significance of these interactions as determined by ANOVA (Table 3) [55,56].

#### 3.2.2. Validation Results

Response surface optimization (Design-Expert^®^ 13.0) identified the ideal preparation parameters as 0.061% *w*/*v* Nano-SiO_2_, 1.822% *w*/*v* pullulan, and 0.774% *w*/*v* glycerol, yielding a predicted overall performance score (K) of 0.780. After adjustment for operational feasibility, validation experiments (*n* = 3) achieved K = 0.777 ± 0.005, showing a mean absolute deviation of 0.25% ± 0.04% from predicted values—confirming the high accuracy of the model. The optimized Nano-SiO_2_/TP/PUL films demonstrated enhanced functional properties: WVP reached 0.2063 g·mm·m^−2^·h^−1^·kPa^−1^, tensile strength (TS) was 2.62 MPa, and elongation at break (EB) was 67.67%. The successful development of high-performance edible films through response surface optimization is of considerable practical importance, particularly when such films exhibit superior mechanical and barrier properties [53].

### 3.3. Analysis of Functional Characteristics of Composite Films

#### 3.3.1. Analysis of Antioxidant Properties

**Figure 2:**
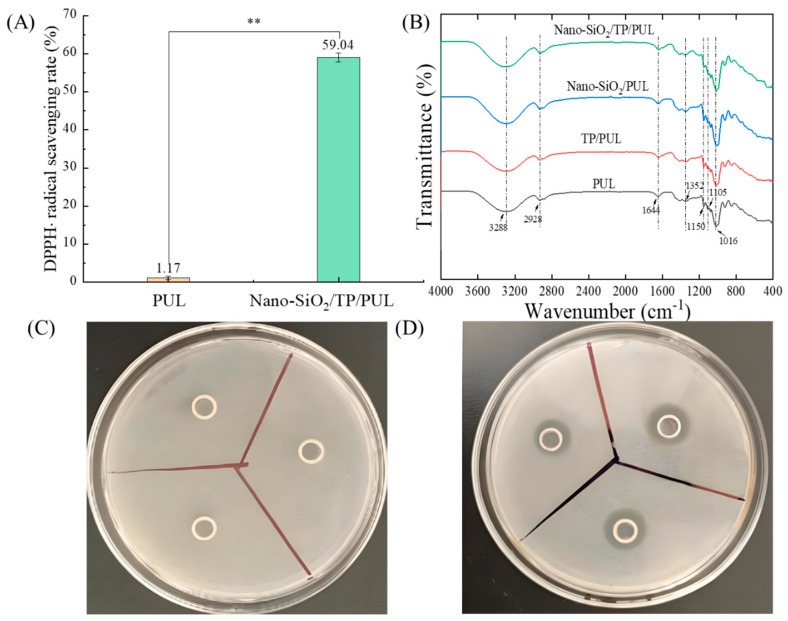
Functional characteristics of composite films. (**A**) represents DPPH·scavenging rate, (**B**) represents FTIR spectra. (**C**,**D**) represent the antibacterial properties of the PUL and Nano-SiO_2_/TP/PUL films against *Staphylococcus aureus.* ** indicates that the difference is very significant, *p* < 0.01.

DPPH radical scavenging capacity, a primary metric for evaluating antioxidant functionality, was significantly enhanced in the composite films [1]. As shown in Figure 2A, control films exhibited negligible scavenging activity (1.17 ± 0.15%), which can be attributed to the limited hydrogen-donating capacity of pullulan hydroxyl groups. In contrast, the optimal Nano-SiO_2_/TP/PUL formulation achieved a markedly higher scavenging rate of 59.04 ± 0.12% (*p* < 0.01). This enhancement can be primarily attributed to the chain-breaking antioxidant mechanism of tea polyphenols, in which catechol groups facilitate hydrogen dissociation through extended π-conjugation, enabling efficient radical neutralization via proton-coupled electron transfer [29,57]. This study focused on the performance of the final composite film; therefore, a direct comparison with TP/PUL films was not conducted.

#### 3.3.2. Antibacterial Assessment

Antibacterial assessment using the Oxford cup method (Figure 2C,D) revealed clear functional differences: pullulan-only solutions produced no detectable inhibition zones against Staphylococcus aureus, whereas the Nano-SiO_2_/TP/PUL composite exhibited significant bacteriostatic activity, with an inhibition zone diameter of 12.3 ± 0.5 mm. The observed antibacterial effects may be attributed to mechanisms such as disruption of cellular metabolism by Nano-SiO_2_ and/or impairment of membrane integrity by TP [54,58]. Future studies will explore these mechanisms in greater detail and expand the assessment to include Gram-negative bacteria and fungi commonly associated with postharvest fruit spoilage. These results highlight the potential practical value of the developed composite as a functional material for next-generation food packaging.

### 3.4. Analysis of Construction Characteristics of Composite Films

#### 3.4.1. SEM

**Figure 3:**
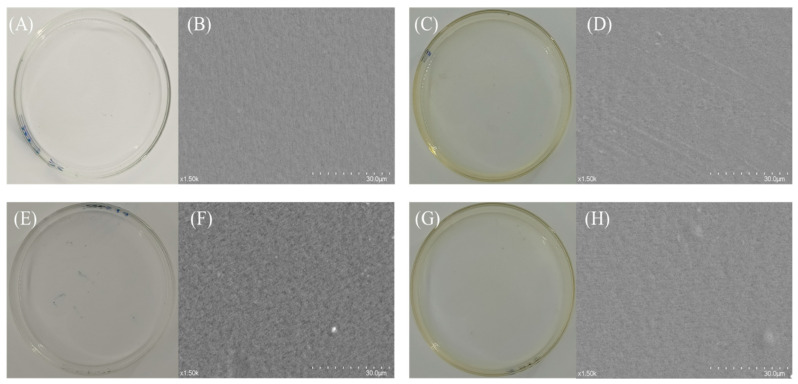
Microstructure of composite films: (**A**,**B**) represent PUL films; (**C**,**D**) represent TP/PUL films; (**E**,**F**) represent Nano-SiO_2_/PUL films; (**G**,**H**) represent Nano-SiO_2_/TP/PUL films. (**A**,**C**,**E**,**G**) are macroscopic images of the composite films, while (**B**,**D**,**F**,**H**) are SEM images of the composite film surfaces.

Scanning electron microscopy (SEM) revealed distinct microstructural differences among the film formulations (Figure 3). Macroscopically, all films exhibited smooth surfaces, with the pure pullulan film appearing colorless and transparent (Figure 3B), while the TP/PUL composite showed a pale-yellow hue (Figure 3D). The Nano-SiO_2_/PUL film displayed noticeable particle agglomeration and increased surface roughness (Figure 3F) [25,48], suggesting inadequate interfacial compatibility. In contrast, the Nano-SiO_2_/TP/pullulan composite exhibited a relatively uniform surface morphology with dense, convex microstructural features under SEM (Figure 3H), indicating the possible formation of nanoscale or submicron architectures. Tea polyphenols likely facilitated the uniform dispersion of Nano-SiO_2_ through hydrogen-bond bridging, promoting the formation of continuous percolation networks that enhanced the structural integrity of the composite—confirming the role of TP as a compatibilizer between silica nanoparticles and the polysaccharide matrix [27].

#### 3.4.2. FTIR

Fourier transform infrared spectroscopy (FTIR) was employed to investigate interfacial interactions, particularly hydrogen bonding, within the composite film in the absence of covalent bonding (Figure 2B). A shift in the O–H stretching vibration from 3288 cm^−1^ to 3303 cm^−1^ indicates the presence of intermolecular hydrogen bonding [10]. Key spectral features included absorption bands at 2928 cm^−1^ (νC–H), 1644 cm^−1^ (νC=O), 1342 cm^−1^ (aromatic νC–O), and 1150 cm^−1^ (glycosidic νC–O). Notable spectral modifications included the following: (1) attenuation of the glycosidic vibration at 1105 cm^−1^, indicating interactions between Nano-SiO_2_ and polysaccharides; (2) a reduction in intensity of the C–O–C stretch at 1016 cm^−1^, suggesting conformational rearrangements induced by tea polyphenols and Nano-SiO_2_. The absence of new absorption bands confirmed that film formation occurred through physical blending without covalent bonding. These spectral changes, which align with previous reports [4,42,59], are consistent with the improved mechanical properties (EB and TS), likely resulting from efficient stress transfer within the composite network.

### 3.5. Application of Composite Coating for Cherry Tomatoes

#### 3.5.1. Effect of Coating on Appearance and Sensory Evaluation

**Figure 4:**
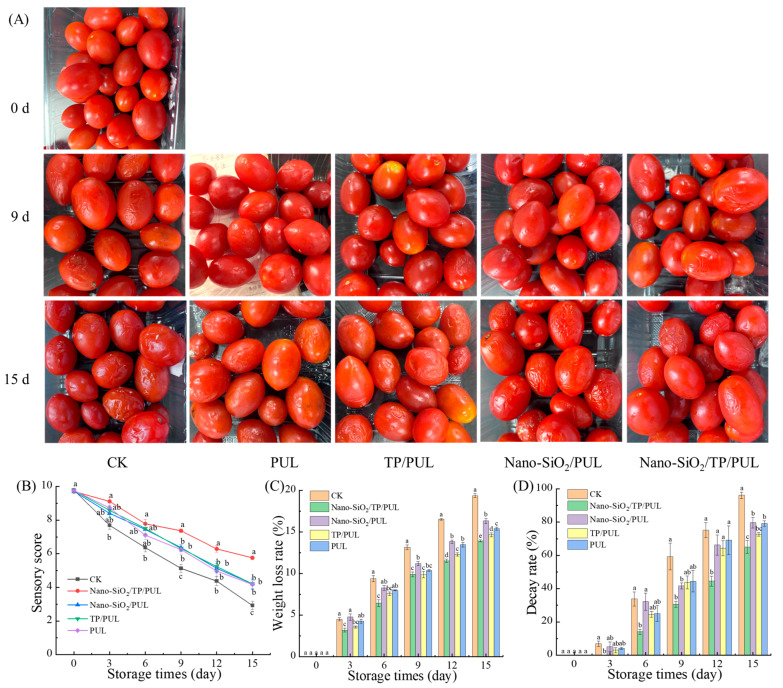
The impact of coating on external quality. (**A**) represents visual effect, (**B**) represents sensory evaluation, (**C**) represents weight loss rate, and (**D**) represents decay rate. Different lowercase superscript letters indicate statistically significant differences (*p* < 0.05) among treatment groups at the same time point, as determined by Tukey’s honestly significant difference (HSD) test.

Sensory evaluation indicated progressive quality deterioration in cherry tomatoes during storage (Figure 4A,B), marked by significant declines in appearance, color, and flavor scores (*p* < 0.05). The control (CK) group exhibited the most rapid quality loss, with sensory ratings 42–96% lower than those of coated groups by day 15 (*p* < 0.05). This decline may be attributed to respiratory nutrient consumption leading to textural changes [60]. By day 9, all coated fruit retained commercial acceptability (scores > 6), whereas the CK group showed visible spoilage (Figure 4B). Notably, fruit coated with the Nano-SiO_2_/TP/PUL composite maintained superior sensory attributes throughout storage. At day 15, the Nano-SiO_2_/TP/PUL-treated fruit received significantly higher scores (5.77) compared to other treatments (*p* < 0.05), exhibiting only severe wrinkling, while the CK fruit developed extensive softening and mold. Other composite coatings showed moderate deterioration but nevertheless outperformed the CK group. The synergistic combination of Nano-SiO_2_, TP, and PUL in the composite matrix overcomes limitations inherent in single-component films, simultaneously enhancing antibacterial efficacy and oxygen barrier properties. This multi-functional improvement significantly boosts the coating’s preservation performance. The Nano-SiO_2_/TP/PUL coating forms a protective barrier that suppresses fruit respiration, and its synergistic formulation mitigates the shortcomings of single-polysaccharide coatings through improved antimicrobial and barrier functions [61]. The shelf-life extension observed in this study is consistent with findings reported by Chen et al. [8], who described a similar effect using a konjac glucomannan/curdlan-based emulsion coating. This agreement suggests that, despite differences in composition, both coatings provide a comparable protective barrier and bioactive environment, which are essential for delaying postharvest deterioration in cherry tomatoes. As a result, the Nano-SiO_2_/TP/PUL composite coating extends the shelf life of cherry tomatoes by 6 days, effectively delaying ripening and senescence.

#### 3.5.2. Effect of Coating on Weight Loss Rate and Decay Rate

Changes in the weight loss rate of cherry tomatoes during storage were primarily due to moisture loss through transpiration and respiratory substrate consumption. As shown in Figure 4C, the cherry tomatoes showed a progressive increase in mass loss throughout storage. The control (CK) group exhibited significantly higher weight loss (18.95% on day 15) than all coated groups (*p* < 0.05). Differences in efficacy were observed among the composite films: the Nano-SiO_2_/TP/PUL-, Nano-SiO_2_/PUL-, TP/PUL-, and PUL-coated groups showed weight losses of 13.72%, 16.33%, 14.87%, and 15.74% at day 15, respectively. The Nano-SiO_2_/TP/PUL coating achieved the highest reduction in weight loss (27.6% compared to CK, *p* < 0.05) by significantly suppressing transpiration and limiting gas exchange. This enhanced performance can be attributed to the synergistic effect of Nano-SiO_2_, TP, and PUL, which compensates for the limitations of single-component films [29,34]. Furthermore, the composite coating formed a semi-permeable barrier on the fruit surface, reducing the penetration of air and moisture while minimizing nutrient loss, thereby enhancing its preservative effect [62]. The Nano-SiO_2_/TP/PUL composite coating was more effective in reducing nutrient loss in cherry tomatoes compared to other formulations.

Decay in cherry tomatoes manifested as surface softening and skin wrinkling, with incidence increasing progressively throughout storage (Figure 4D). This senescence-related deterioration results from loss of cellular integrity, which facilitates enzymatic nutrient breakdown and microbial colonization [63]. By day 3, no decay was observed in the Nano-SiO_2_/TP/PUL-coated group, compared to a 6.84% decay rate in the control (CK) group. By day 15, the CK group exhibited 96.19% decay, significantly higher than all coated groups (*p* < 0.05): Nano-SiO_2_/TP/PUL (65.09%), Nano-SiO_2_/PUL (79.70%), TP/PUL (72.64%), and PUL-only (79.19%). The Nano-SiO_2_/TP/PUL formulation provided the strongest decay suppression (a 31.1% reduction compared to the next-best treatment, *p* < 0.05), attributable to its synergistic antimicrobial mechanism: Nano-SiO_2_ disrupts microbial membranes, while tea polyphenols inhibit extracellular enzyme secretion [64]. These results demonstrate the effectiveness of the Nano-SiO_2_/TP/PUL coating in delaying histological degradation in cherry tomatoes.

#### 3.5.3. Effect of Coating on Respiration Rate

Respiration intensity is directly correlated with the metabolic rate of fruits, serving as a key indicator of their physiological activity, quality changes, and storage potential [65]. As such, monitoring the respiration rate (RR) provides critical insight into the maturation process of cherry tomatoes. As summarized in Table 4, the respiration intensity of cherry tomatoes initially increased, followed by a gradual decline. The control (CK) group reached a maximum respiration intensity of 45.66 mg/kg·h on day 9, after which it decreased, reaching a minimum of 21.96 mg/kg·h on day 15. This pattern is consistent with the behavior of climacteric fruit: after harvest, increased metabolic activity during early maturation leads to a rise in respiratory intensity [7,66]. In contrast, the respiration peaks for cherry tomatoes coated with Nano-SiO_2_/TP/PUL, Nano-SiO_2_/PUL, TP/PUL, and PUL were 35.88 mg/kg·h, 42.77 mg/kg·h, 38.23 mg/kg·h, and 40.73 mg/kg·h on day 9, respectively, decreasing to minimum values of 13.45 mg/kg·h, 18.38 mg/kg·h, 15.76 mg/kg·h, and 15.76 mg/kg·h on day 15. These results indicate that the coating treatments effectively reduced the respiration rate, resulting in significantly lower respiration peaks (*p* < 0.05). This finding aligns with previous research by Zhang et al. [67], who reported that a chitosan-based composite coating suppressed the exchange of CO_2_ and O_2_ in sweet cherries, thereby reducing decay. The observed suppression of respiration is likely due to the modified atmosphere created by the coating. The film forms a semi-permeable barrier that encloses the fruit in a confined microenvironment. As respiration continues, O_2_ levels decrease due to consumption and CO_2_ accumulates, leading to an atmosphere that inhibits respiratory metabolism and delays ripening and senescence [65]. Among all treatments, the Nano-SiO_2_/TP/PUL composite film exhibited the strongest inhibitory effect on the respiration rate of cherry tomatoes.

#### 3.5.4. Effect of Coating on the Firmness and VC

Fruit firmness is primarily governed by the structural integrity and composition of cell wall components, particularly pectin and cellulose [16]. As shown in Table 4, firmness decreased progressively during storage, due to factors such as moisture evaporation, cell wall relaxation, and enzymatic degradation of pectin by pectinase [68,69]. Significant differences (*p* < 0.05) in firmness retention were observed among treatments. The control (CK) group showed the most pronounced decline, reaching 0.54 N at day 15, while the coated groups exhibited better retention: 2.40 N (Nano-SiO_2_/TP/PUL), 1.39 N (Nano-SiO_2_/PUL), 1.72 N (TP/PUL), and 1.18 N (PUL). The superior performance of the Nano-SiO_2_/TP/PUL coating is attributed to its optimized barrier properties—specifically its low water vapor permeability (WVP: 0.2063 g·mm·m^−2^·h^−1^·kPa^−1^) and improved mechanical strength—which collectively reduce moisture loss and physical damage, thereby delaying pectinase-mediated pectin degradation and subsequent softening [70]. All composite coatings significantly delayed softening compared to the control (*p* < 0.05). These results demonstrate that the Nano-SiO_2_/TP/PUL coating most effectively inhibits firmness loss in cherry tomatoes.

Vitamin C (VC), an essential nutrient known for its antioxidant properties and role in enhancing iron absorption, serves as a key indicator of fruit nutritional quality [71]. As shown in Table 4, VC content decreased progressively during storage, primarily due to respiratory consumption and enzymatic oxidation mediated by endogenous fruit enzymes [29]. The control (CK) group showed the most pronounced reduction, declining from 39.57 mg/100 g to 16.57 mg/100 g. All coating treatments exhibited significantly higher VC retention (*p* < 0.05) compared to the CK group. After 15 days, VC content in the Nano-SiO_2_/TP/PUL, Nano-SiO_2_/PUL, TP/PUL, and PUL groups exceeded that of the CK group by 49.53%, 29.88%, 43.23%, and 36.34%, respectively. This improvement can be attributed to the functional characteristics of the coating: the antioxidant activity (59.04% DPPH scavenging capacity) provided by TP effectively inhibited VC oxidation, while the dense, homogeneous structure (as evidenced by SEM and FTIR analyses) limited oxygen permeability, thereby reducing enzymatic degradation [72]. Overall, these findings demonstrate that the Nano-SiO_2_/TP/PUL composite film provides the most effective protection against VC degradation in cherry tomatoes.

#### 3.5.5. Effect of Coating on the TSS and TA

Total soluble solids (TSS), a key indicator of fruit quality representing the total content of water-soluble compounds such as sugars, acids, vitamins, and minerals [66], showed a characteristic biphasic pattern during storage of cherry tomatoes (Table 4). An initial increase in TSS was caused by the hydrolysis of starch into soluble sugars, while the subsequent decrease resulted from respiratory consumption of substrates and microbial degradation [73,74]. The low WVP of the coating effectively minimized moisture loss, and the reduced oxygen permeability—attributed to the dense Nano-SiO_2_/TP/PUL network—suppressed respiratory intensity, thereby slowing the consumption of sugars and acids as metabolic substrates [66]. The composite coatings significantly mitigated TSS fluctuations (*p* < 0.05), reducing the peak value observed on day 9 and delaying the decline in the later storage period. After 15 days, coated tomatoes retained 48.58% higher TSS levels than the CK group. This stabilization reflects the inhibited decomposition of organic acids and polysaccharides during respiratory metabolism [57]. Notably, the Nano-SiO_2_/TP/PUL coating most effectively minimized TSS variation among all treatments.

The decrease in titratable acidity (TA) during storage of cherry tomatoes (Table 4) is mainly due to increased respiratory metabolism, which consumes organic acids as substrates, along with potential biochemical conversions of acidic compounds [75]. The improved TA retention, especially in the Nano-SiO_2_/TP/PUL group, can be explained by the multifunctional nature of the coating: its oxygen barrier properties likely delayed the aerobic respiration responsible for organic acid consumption, while the antioxidant activity from tea polyphenols may have reduced acid oxidation, collectively contributing to better acid retention [21]. The control (CK) group showed a pronounced decline, reaching a minimum TA of 0.312%, whereas all coated groups exhibited significantly slower rates of reduction. After 15 days, TA retention in the Nano-SiO_2_/TP/PUL, Nano-SiO_2_/PUL, TP/PUL, and PUL groups exceeded the CK group by 8.97%, 3.53%, 6.73%, and 5.12%, respectively. This enhanced retention confirms that the composite coatings effectively inhibit the decomposition of organic acids and help stabilize TA levels [34]. Importantly, the Nano-SiO_2_/TP/PUL coating performed better than all other treatments in reducing TA loss.

### 3.6. PCA of Coating Effects

**Figure 5:**
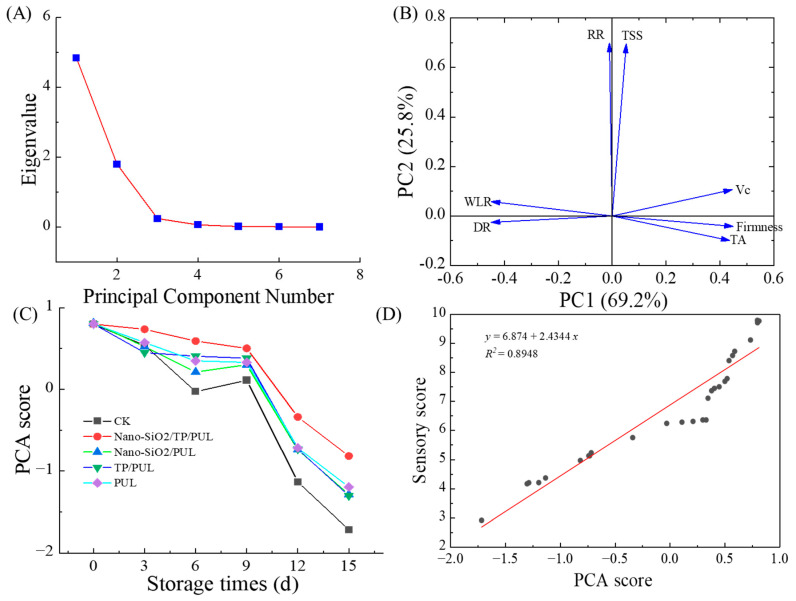
Result of PCA analysis. (**A**) is the feature vector value plot, (**B**) is the loading plot, (**C**) is the PCA score plot, and (**D**) represents the plot of the linear fit between the sensory score and the PCA score.

Principal component analysis (PCA) was performed on seven quality indices of cherry tomatoes—firmness, weight loss rate, decay rate, titratable acidity, vitamin C, respiration rate, and total soluble solids—to minimize subjective bias and avoid overreliance on any single metric. The initial Kaiser–Meyer–Olkin (KMO) value (0.792, exceeding the 0.6 threshold) and Bartlett’s test of sphericity (χ^2^ = 464.90, *p* < 0.005) confirmed the suitability of the data for PCA [69]. Two principal components (PCs) with eigenvalues greater than 1 were extracted, collectively accounting for 94.95% of the total variance (Figure 5A). PC1, which included firmness, weight loss rate, decay rate, vitamin C, and titratable acidity, represented physical integrity, nutritional quality, and stress resistance. PC2, composed of respiration rate and total soluble solids, reflected physiological metabolism and sensory attributes (Figure 5B). Standardized linear expressions for PC1 and PC2 (Equations (13) and (14)) were derived based on eigenvector coefficients, and the composite PCA score was calculated using Equation (15).

*F*_1_ = −0.169 ∗ *Z_WLR_* − 0.171 ∗ *Z_DR_* + 0.170 ∗ *Z_firmness_* + 0.013 ∗ *Z_TSS_* + 0.164 ∗ *Z_TA_* + 0.172 ∗ *Z_VC_* + 0.013 ∗ *Z_RR_* (13)

*F*_2_ = 0.013 ∗ *Z_WLR_* − 0.301 ∗ *Z_DR_* − 0.014 ∗ *Z_firmness_* + 0.553 ∗ *Z_TSS_* − 0.076 ∗ *Z_TA_* + 0.096 ∗ *Z_VC_* + 0.536 ∗ *Z_RR_* (14)

*F* = (0.6919 *F*_1_ + 0.2575 *F*_2_)/0.9495 (15)

As storage time increased, PCA composite scores gradually decreased; however, coating treatments slowed this decline, with the most pronounced effect observed in the Nano-SiO_2_/TP/PUL-coated group (Figure 5C). A strong linear correlation was identified between PCA scores and sensory evaluation scores: y = 6.874 + 2.4344x (R^2^ = 0.8948, *p* < 0.01; Figure 5D). This highly significant correlation confirms that PCA can effectively serve as a comprehensive substitute for individual quality metrics and demonstrates that the coatings effectively delay quality deterioration [76]. Furthermore, analysis of variable loadings within the PCA model indicated that firmness, vitamin C (VC) content, decay incidence, and weight loss rate were the primary factors influencing consumer acceptability. The enhanced ability of the Nano-SiO_2_/TP/PUL coating to preserve these key attributes, as demonstrated in previous analyses, is directly correlated with its superior performance in maintaining sensory quality and delaying consumer rejection [77,78]. This preservation effect became increasingly evident during the mid-to-late storage periods, with the Nano-SiO_2_/TP/PUL coating consistently exhibiting the best performance among all treatments.

## 4. Conclusions

An edible composite film was successfully developed using response surface methodology. The optimal concentrations were determined as 0.06% Nano-SiO_2_, 0.1% tea polyphenols (TP), 1.8% pullulan (PUL), and 0.77% glycerol (*w*/*v*). The resulting film demonstrated promising functional characteristics, including a water vapor permeability of 0.2063 g·mm·m^−2^·h^−1^·kPa^−1^, a tensile strength of 2.62 MPa, and an elongation at break of 67.67%, as well as detectable antioxidant and antibacterial activities. Microscopic analysis indicated a homogeneous microstructure. When applied as a coating on cherry tomatoes, the treatment led to a significant improvement (*p* < 0.05) in postharvest quality relative to the uncoated control. Specifically, the coating enhanced firmness retention, reduced the loss of titratable acidity by 18.7% and vitamin C by 22.4%, decreased weight loss (1.8% vs. 3.2% in the control at day 9), lowered decay incidence (by 34%), moderated changes in total soluble solids (TSS), and suppressed respiration rate. These positive effects collectively resulted in a shelf-life extension of 6 days under the applied experimental conditions. Multivariate principal component analysis (PCA) further confirmed the effectiveness of the coating in maintaining overall fruit quality.

For future work, a comprehensive safety and regulatory assessment of Nano-SiO_2_ in edible coatings should be conducted, considering its current status as an approved food additive (e.g., E551 in the EU and under FDA regulations). Additional studies are needed to systematically evaluate economic feasibility—such as large-scale production costs—and functional performance under diverse, realistic storage conditions, as well as across various types of produce, to facilitate potential commercial adoption.

## Figures and Tables

**Table 1:** Effects of processing parameters on mechanical and physical properties of Nano-SiO_2_/TP/PUL composite films.

Factors	LT (%)	WVP (g ∗ mm/(m^2^ ∗ h ∗ Kpa))	TS (Mpa)	EB (%)	K
Nano-SiO_2_ (g)					
0.02	93.43 ± 2.35 ^a^	0.62 ± 0.12 ^a^	1.25 ± 0.03 ^c^	29.33 ± 3.12 ^c^	0.16 ± 0.05 ^c^
0.04	90.09 ± 1.78 ^a^	0.46 ± 0.07 ^b^	2.13 ± 0.12 ^b^	31.67 ± 4.01 ^c^	0.47 ± 0.03 ^b^
0.06	87.56 ± 2.36 ^b^	0.38 ± 0.06 ^b^	3.03 ± 0.32 ^a^	44.67 ± 2.35 ^b^	0.64 ± 0.03 ^a^
0.08	81.72 ± 3.52 ^c^	0.28 ± 0.05 ^c^	2.73 ± 0.25 ^a^	47.67 ± 3.81 ^b^	0.60 ± 0.05 ^a^
0.10	83.52 ± 1.26 ^c^	0.31 ± 0.08 ^c^	2.07 ± 0.16 ^b^	59.67 ± 2.36 ^a^	0.61 ± 0.06 ^a^
PUL (g)					
1.50	82.97 ± 1.44 ^b^	0.28 ± 0.05 ^b^	2.64 ± 0.12 ^b^	70.67 ± 2.36 ^a^	0.67 ± 0.05 ^ab^
1.75	85.70 ± 0.14 ^ab^	0.17 ± 0.06 ^c^	3.3 ± 0.25 ^a^	57 ± 3.52 ^ab^	0.72 ± 0.04 ^a^
2.00	86.07 ± 2.92 ^ab^	0.22 ± 0.07 ^c^	2.75 ± 0.08 ^b^	47.67 ± 4.13 ^b^	0.61 ± 0.01 ^b^
2.25	89.33 ± 1.33 ^a^	0.40 ± 0.02 ^c^	2.58 ± 0.05 ^b^	39.73 ± 3.25 ^bc^	0.46 ± 0.07 ^c^
2.50	87.50 ± 2.56 ^a^	0.55 ± 0.07 ^a^	1.98 ± 0.23 ^c^	24 ± 4.69 ^c^	0.22 ± 0.03 ^d^
TP (g)					
0.00	90.14 ± 1.56 ^a^	0.64 ± 0.07 ^a^	1.76 ± 0.25 ^b^	22.33 ± 2.13 d	0.13 ± 0.03 b
0.05	87.60 ± 2.36 ^ab^	0.27 ± 0.01 ^c^	2.61 ± 0.19 ^a^	42.01 ± 0.86 ^b^	0.55 ± 0.06 ^a^
0.10	89.03 ± 1.56 ^ab^	0.22 ± 0.04 ^c^	2.75 ± 0.08 ^a^	55.33 ± 3.25 ^a^	0.63 ± 0.05 ^a^
0.15	86.93 ± 0.86 ^ab^	0.18 ± 0.08 ^c^	1.33 ± 0.13 ^c^	33.67 ± 2.32 ^c^	0.50 ± 0.03 ^a^
0.20	86.51 ± 1.23 ^b^	0.51 ± 0.04 ^b^	1.16 ± 0.05 ^d^	26.67 ± 3.26 ^d^	0.21 ± 0.07 ^b^
Glycerol (g)					
0.40	81.28 ± 2.56 ^d^	0.41 ± 0.06 ^b^	7.46 ± 1.23 ^a^	28.67 ± 2.18 ^c^	0.58 ± 0.03 ^b^
0.60	87.77 ± 1.32 ^b^	0.29 ± 0.04 ^bc^	4.42 ± 0.56 ^b^	35.67 ± 3.25 ^c^	0.57 ± 0.03 ^b^
0.80	92.10 ± 0.69 ^a^	0.25 ± 0.01 ^c^	2.72 ± 0.68 ^c^	52.33 ± 6.23 ^b^	0.64 ± 0.01 ^a^
1.00	90.02 ± 1.96 ^ab^	0.27 ± 0.06 ^bc^	1.27 ± 0.32 ^d^	57.33 ± 7.2 ^b^	0.57 ± 0.06 ^b^
1.20	87.40 ± 1.35 ^c^	0.75 ± 0.08 ^a^	0.77 ± 0.67 ^d^	74.33 ± 4.39 ^a^	0.30 ± 0.06 ^c^

Within each column, means sharing the same superscript letter are not significantly different across factor levels (*p* < 0.05). LT, WVP, TS, EB, and K denote light transmittance, water vapor permeability, tensile strength, elongation at break, and comprehensive performance score, respectively. Data are means ± SD.

**Table 2:** Design and results of response surface test.

Run	Nano-SiO_2_ (%, *m*/*v*)	PUL (%, *m*/*v*)	Glycerol (%, *m*/*v*)	K
1	−1 (0.04)	−1 (1.50)	0 (0.80)	0.39 ± 0.02
2	1 (0.08)	−1 (1.50)	0 (0.80)	0.48 ± 0.03
3	−1 (0.04)	1 (2.00)	0 (0.80)	0.6 ± 0.03
4	1 (0.08)	1 (2.00)	0 (0.80)	0.58 ± 0.05
5	−1 (0.04)	0 (1.75)	−1 (0.60)	0.6 ± 0.03
6	1 (0.08)	0 (1.75)	−1 (0.60)	0.53 ± 0.05
7	−1 (0.04)	0 (1.75)	1 (1.00)	0.31 ± 0.01
8	1 (0.08)	0 (1.75)	1 (1.00)	0.49 ± 0.02
9	0 (0.06)	−1 (1.50)	−1 (0.60)	0.54 ± 0.03
10	0 (0.06)	1 (2.00)	−1 (0.60)	0.54 ± 0.04
11	0 (0.06)	−1 (1.50)	1 (1.00)	0.19 ± 0.03
12	0 (0.06)	1 (2.00)	1 (1.00)	0.61 ± 0.02
13	0 (0.06)	0 (1.75)	0 (0.80)	0.79 ± 0.03
14	0 (0.06)	0 (1.75)	0 (0.80)	0.75 ± 0.05
15	0 (0.06)	0 (1.75)	0 (0.80)	0.76 ± 0.04
16	0 (0.06)	0 (1.75)	0 (0.8)	0.75 ± 0.03
17	0 (0.06)	0 (1.75)	0 (0.8)	0.74 ± 0.02

Note: −1, 0, and 1 represent low, medium, and high levels, respectively.

**Table 3:** Analysis of variance for the response surface model.

Source	Sum of Squares	df	Mean Square	F-Value	*p*-Value	Significant
Model	0.4012	9	0.0446	74.55	<0.0001	**
A-Nano SiO_2_	0.0045	1	0.0045	7.49	0.0291	*
B-PUL	0.0551	1	0.0551	92.07	<0.0001	**
C-Glycerol	0.0358	1	0.0358	59.87	0.0001	**
AC	0.0162	1	0.0162	27.16	0.0012	**
BC	0.0307	1	0.0307	51.30	0.0002	**
A^2^	0.0668	1	0.0668	111.68	<0.0001	**
B^2^	0.0637	1	0.0637	106.60	<0.0001	**
C^2^	0.0983	1	0.0983	164.43	<0.0001	**
Residual	0.0042	7	0.0006			
Lack of Fit	0.0024	3	0.0008	1.80	0.2865	
Pure Error	0.0018	4	0.0004			
Cor Total	0.4054	16				

Note: ** indicates that the difference is very significant, *p* < 0.01. * indicates a significant difference, *p* < 0.05.

**Table 4:** Quality changes of cherry tomatoes during storage.

Indices	Treatments	Storage Times (Days)					
		0 d	3 d	6 d	9 d	12 d	15 d
Firmness	CK	11.85 ± 0.29 ^a^	9.69 ± 0.36 ^b^	5.32 ± 0.14 ^c^	2.73 ± 0.41 ^c^	1.52 ± 0.07 ^d^	0.54 ± 0.10 ^d^
	Nano-SiO_2_/TP/PUL	11.85 ± 0.43 ^a^	10.43 ± 0.25 ^a^	8.39 ± 0.32 ^a^	6.20 ± 0.22 ^a^	4.47 ± 0.43 ^a^	2.40 ± 0.15 ^a^
	Nano-SiO_2_/PUL	11.85 ± 0.19 ^a^	8.85 ± 0.379 ^c^	6.78 ± 0.34 ^b^	4.91 ± 0.37 ^b^	2.34 ± 0.57 ^c^	1.39 ± 0.22 ^bc^
	TP/PUL	11.85 ± 0.04 ^a^	9.62 ± 0.077 ^b^	7.50 ± 0.41 ^ab^	5.83 ± 0.24 ^a^	3.20 ± 0.18 ^b^	1.72 ± 0.21 ^b^
	PUL	11.85 ± 0.42 ^a^	8.29 ± 0.34 ^d^	7.32 ± 0.42 ^ab^	4.89 ± 0.31 ^b^	3.05 ± 0.15 ^b^	1.18 ± 0.25 ^c^
TSS	CK	6.66 ± 0.21 ^a^	7.17 ± 0.10 ^d^	8.83 ± 0.07 ^a^	10.53 ± 0.15 ^a^	6.34 ± 0.11 ^e^	5.27 ± 0.06 ^d^
	Nano-SiO_2_/TP/PUL	6.65 ± 0.22 ^a^	7.73 ± 0.10 ^a^	8.69 ± 0.12 ^b^	9.35 ± 0.11 ^c^	8.32 ± 0.09 ^a^	7.83 ± 0.04 ^a^
	Nano-SiO_2_/PUL	6.73 ± 0.16 ^a^	7.50 ± 0.06 ^b^	8.21 ± 0.03 ^d^	9.62 ± 0.16 ^b^	7.38 ± 0.05 ^b^	6.27 ± 0.06 ^b^
	TP/PUL	6.65 ± 0.05 ^a^	7.35 ± 0.02 ^c^	8.22 ± 0.05 ^d^	9.02 ± 0.06 ^d^	6.91 ± 0.05 ^d^	5.75 ± 0.08 ^c^
	PUL	6.66 ± 0.10 ^a^	7.29 ± 0.07 ^c^	8.48 ± 0.05 ^c^	9.61 ± 0.07 ^b^	7.14 ± 0.11 ^c^	5.65 ± 0.10 ^c^
TA	CK	0.38 ± 0.01 ^a^	0.37 ± 0.01 ^a^	0.33 ± 0.06 ^c^	0.32 ± 0.00 ^d^	0.31 ± 0.01 ^c^	0.31 ± 0.00 ^c^
	Nano-SiO_2_/TP/PUL	0.38 ± 0.01 ^a^	0.37 ± 0.01 ^a^	0.36 ± 0.00 ^a^	0.36 ± 0.01 ^a^	0.35 ± 0.01 ^a^	0.34 ± 0.00 ^a^
	Nano-SiO_2_/PUL	0.38 ± 0.01 ^a^	0.37 ± 0.01 ^a^	0.34 ± 0.05 ^bc^	0.33 ± 0.01 ^c^	0.33 ± 0.01 ^b^	0.32 ± 0.00 ^b^
	TP/PUL	0.38 ± 0.01 ^a^	0.37 ± 0.01 ^a^	0.35 ± 0.05 ^ab^	0.35 ± 0.01 ^ab^	0.33 ± 0.01 ^b^	0.33 ± 0.01 ^b^
	PUL	0.38 ± 0.01 ^a^	0.37 ± 0.00 ^a^	0.36 ± 0.05 ^a^	0.35 ± 0.01 ^ab^	0.33 ± 0.00 ^b^	0.33 ± 0.00 ^b^
VC	CK	39.57 ± 0.09 ^b^	34.66 ± 0.11 ^e^	31.34 ± 0.20 ^d^	28.54 ± 0.11 ^d^	20.64 ± 0.20 ^e^	16.57 ± 0.22 ^e^
	Nano-SiO_2_/TP/PUL	39.63 ± 0.17 ^ab^	38.46 ± 0.11 ^a^	35.77 ± 0.18 ^a^	33.68 ± 0.21 ^a^	27.71 ± 0.17 ^a^	24.79 ± 0.10 ^a^
	Nano-SiO_2_/PUL	39.85 ± 0.19 ^a^	37.52 ± 0.10 ^c^	34.73 ± 0.21 ^b^	31.64 ± 0.12 ^c^	25.45 ± 0.22 ^c^	21.53 ± 0.16 ^d^
	TP/PUL	39.76 ± 0.11 ^ab^	37.82 ± 0.14 ^b^	34.25 ± 0.23 ^c^	32.23 ± 0.09 ^b^	26.34 ± 0.17 ^b^	23.74 ± 0.16 ^b^
	PUL	39.62 ± 0.06 ^b^	35.50 ± 0.14 ^d^	34.11 ± 0.05 ^c^	31.65 ± 0.21 ^c^	24.77 ± 0.12 ^d^	22.60 ± 0.14 ^c^
RR	CK	22.00 ± 0.33 ^a^	26.82 ± 0.45 ^a^	33.89 ± 0.34 ^c^	45.66 ± 0.34 ^a^	30.03 ± 0.58 ^a^	21.96 ± 0.66 ^a^
	Nano-SiO_2_/TP/PUL	22.06 ± 0.34 ^a^	23.13 ± 0.36 ^b^	28.18 ± 0.37 ^b^	35.88 ± 0.11 ^e^	16.33 ± 0.42 ^e^	13.45 ± 0.46 ^d^
	Nano-SiO_2_/PUL	22.00 ± 0.23 ^a^	20.89 ± 0.23 ^cd^	28.86 ± 0.65 ^ab^	42.77 ± 00.20 ^b^	23.75 ± 0.13 ^c^	18.38 ± 0.15 ^b^
	TP/PUL	22.06 ± 0.44 ^a^	20.47 ± 0.49 ^d^	29.46 ± 0.41 ^a^	38.23 ± 0.31 ^d^	22.51 ± 0.49 ^d^	15.76 ± 0.20 ^c^
	PUL	22.07 ± 0.37 ^a^	21.36 ± 0.26 ^c^	29.11 ± 0.29 ^a^	40.73 ± 0.17 ^c^	25.76 ± 0.36 ^b^	16.06 ± 0.16 ^c^

Notes: TSS represents the total soluble solids, TA denotes the titratable acid, and RR signifies the respiration rate. Different lowercase superscript letters within the same column indicate statistically significant differences (*p* < 0.05) among treatments at the same storage time, as determined by Tukey’s honestly significant difference (HSD) test.

## Data Availability

The original contributions presented in the study are included in the article, further inquiries can be directed to the corresponding author.

## Abbreviations

The following abbreviations are used in this manuscript:

ATR-FTIR	Attenuated Total Reflectance-Fourier Transform Infrared Spectroscopy
CK	Control check
DCPIP	2,6-Dichlorophenol Indophenol
DPPH	1,1-Diphenylsulfonylbenzhydrazide
DR	Decay Rate
EB	Elongation at Break
LT	Light Transmittance
Nanno-SiO_2_	Nanoscale Silicon Dioxide
PCA	Principal Component Analysis
PUL	Pullulan
PVA	Polyvinyl Alcohol
RR	Respiration Rate
SEM	Scanning Electron Microscopy
TA	Titratable Acid
TP	Tea Polyphenols
TS	Tensile Strength
TSS	Total Soluble Solids
Vitamin C	VC
WLR	Water Loss Rate
WVP	Water Vapor Permeability
ZnO-Nps	Nanoscale of Zinc Oxide

## Appendix A

**Table A1:** Grouping and treatment of cherry tomatoes.

Groups	Treatment Composition (*w*/*v*)
Nano-SiO_2_/TP/PUL	0.06% Nano-SiO_2_ + 0.1% TP + 1.8% PUL coating solution
Nano-SiO_2_/PUL	0.06% Nano-SiO_2_ + 1.8% PUL coating solution
TP/PUL	0.1% TP + 1.8% PUL coating solution
PUL	1.8% PUL coating solution
CK	Distilled water (uncoated group)

**Table A2:** Scoring criteria for sensory quality of cherry tomatoes.

Score Range	Appearance	Odor	Texture	Decay Degree
8.1–10.0	Vibrant color with gloss	Intense fruity fragrance	Firm	No decay
6.1–8.0	Slight discoloration, partial dehydration	Mild fruity notes	Moderately firm	No visible decay
4.1–6.0	Darkened color, significant dehydration	Slight sour/rotten odor	Softened, non-elastic	Minor decay
2.1–4.0	Dull color, severe wrinkles	Alcoholic off-odor	Spoiled	Severe decay
0–2.0	Blackened appearance	Strong alcoholic odor	Severely mushy	Fully decayed
